# The Effects of Noncompliance to Prolia (Denosumab) on the Changes in Bone Mineral Density: A Retrospective Review

**DOI:** 10.1155/2016/7903128

**Published:** 2016-01-04

**Authors:** Matthew Wong-Pack, Aashish Kalani, Jacob Hordyk, George Ioannidis, Robert Bensen, William G. Bensen, Alexandra Papaioannou, Jonathan D. Adachi, Arthur N. Lau

**Affiliations:** ^1^Faculty of Health Sciences, McMaster University, 501-25 Charlton Avenue E., Hamilton, ON, Canada L8N 1Y2; ^2^University of Ottawa, 75 Laurier Avenue E., Ottawa, ON, Canada K1N 6N5; ^3^Division of Rheumatology, Department of Medicine, McMaster University, 501-25 Charlton Avenue E., Hamilton, ON, Canada L8N 1Y2; ^4^Rheumatology Health Team, St. Joseph's Hospital, 203-25 Charlton Avenue E., Hamilton, ON, Canada L8N 1Y2; ^5^Division of Geriatrics, Department of Medicine, McMaster University, St. Peter's Hospital, HHS 88 Maplewood Avenue, Hamilton, ON, Canada L8M 1W9

## Abstract

Although denosumab (Prolia) has been shown to be a safe and efficacious therapy for osteoporotic patients in numerous clinical trials, few studies have determined its effectiveness in real world clinical practice. A retrospective review of patients prescribed Prolia assessing the impact that noncompliance from the regular dosing regimen of six months for denosumab has on bone mineral density (BMD) was performed. 924 patient records were reviewed between August 2012 and September 2013 with 436 patients meeting the eligibility criteria. Patients were divided into three groups: subsequent injection of denosumab (1) less than five months, (2) between five and seven months, and (3) more than seven months after their initial subcutaneous injection. A multivariable regression analysis was conducted comparing the differences among the three prespecified groups in BMD change (g/cm^2^) after one year of denosumab therapy at both the lumbar spine (LS) and femoral neck (FN). The differences in LS and FN BMD have shown that the relationship between the timing of drug administration in these three groups and change in BMD over 1 year was not clinically or statistically significant (*p* > 0.05). A follow-up study with a larger sample size and longer follow-up duration is required to further characterize this relationship.

## 1. Introduction

Osteoporosis is among the ten most important and prevalent chronic diseases [1, 2] affecting approximately 200 million people worldwide. Its prevalence continues to increase among the elderly population [3]. Osteoporosis is characterized by a progressive decrease in bone mass and microarchitectural deterioration, resulting in increased bone fragility and ultimately an increased risk of fracture [4–6]. Osteoporotic fractures, especially hip and vertebral fractures, are associated with significant morbidity and mortality in the elderly and also pose a significant burden on the health-care system. As the population worldwide continues to age, the prevalence of osteoporosis will continue to climb, with a growing need for effective therapies to prevent fractures [1, 2].

Oral bisphosphonates are the most commonly prescribed class of medications for osteoporosis. Bisphosphonates have been shown to effectively reduce the risk of vertebral and hip fractures in osteoporotic patients. Unfortunately, several large studies have found that the majority of postmenopausal women stop bisphosphonate therapy within 1 year of beginning treatment [7–9]. Reasons for poor persistence include conflicting patient beliefs, patient preferences, financial limitations, and previous adverse effects including rash, myalgia, nausea, and diarrhea [7, 10, 11]. Impaired compliance and persistence to long-term bisphosphonate treatment can ultimately lead to an increased risk of fracture [7].

Denosumab is a fully human monoclonal antibody against RANK ligand used in the treatment of osteoporosis [11]. In osteoporosis, the thinning and increased porosity of cortical bone and disruption of cortical and trabecular architecture is the result of an imbalance in bone remodeling where the rate of bone resorption exceeds bone formation via changes in the RANK/RANK ligand pathway, an important regulator of osteoclast activity [12–17]. In postmenopausal women, increases in RANK ligand production have been associated with an increase in osteoclast activity and overall net bone resorption [18, 19]. Denosumab has an affinity and specificity for RANK ligand and is responsible for inhibiting the proliferation and maturation of osteoclast precursors and functioning of mature osteoclasts [20]. Denosumab has been found to increase bone mineral density (BMD) annually and decrease bone turnover markers for at least six months after injection. Clinical trials have demonstrated that denosumab treatment given subcutaneously once every six months is well tolerated and results in significant decreases in hip, nonvertebral, and vertebral fracture risk [21, 22].

Adherence to antiresorptive agents is crucial to achieve optimal therapeutic results. The objective of this study is to assess the effectiveness of denosumab therapy in the real world setting and the impact that noncompliance with the regular dosing regimen has on BMD (measured at the lumbar spine [LS] and femoral neck [FN]) compared to patients who receive their scheduled dosing regimen.

## 2. Materials and Methods

A retrospective chart review was performed in a single academic practice. Approval by the Hamilton Integrated Research Ethics Board (HIREB) was obtained. All patients from the Charlton Centre for Specialized Treatments in Hamilton, Ontario, Canada, on denosumab were screened for eligibility in the review. A retrospective cohort study was performed from August 2012 to December 2013 for all osteoporotic patients who received a minimum of two subcutaneous injections of denosumab since May 2010. A total of 924 patient records were reviewed with 436 patients fulfilling eligibility criteria. Patients were eligible for this inclusion if they were above 50 years of age with or without prevalent fractures, received at least two subcutaneous injections of denosumab, and had at least two sequential BMD measurements performed one year apart. Patients were excluded if they had a subsequent BMD measurement performed more than one year before or after the initial injection of denosumab, a change in BMD DXA-measurement machine on subsequent BMD measurements, or if they had a prevalent fracture in the femoral neck and/or lumbar spine during the review period, thus making the results incomparable. Included patients were treatment naïve (46) or have previously been treated with oral bisphosphonates [risedronate (210), etidronate (4), and alendronate (95)], the intravenous bisphosphonate [zoledronic acid (29)], raloxifene (29), or teriparatide (23).

Patients were allocated to one of three groups based on their compliance to the product monograph of injections given every 6 months: subsequent injection of denosumab (1) less than five months, (2) between five and seven months, and (3) more than seven months after their initial subcutaneous injection. Compliance was determined by calculating time elapsed between the initial and subsequent injection of denosumab. Patients were considered to be compliant if the two injections of denosumab were 6 months +/− 4 weeks apart. Prevalent and incident fracture data was obtained from the patient's medical records. Fractures were considered to be incident if they occurred during the time frame of this retrospective review.

Baseline characteristics were summarized using descriptive statistics. Formal statistical analyses were conducted using SAS Version 9.1. Multivariable regression analyses were performed to evaluate the differences among the three prespecified groups in BMD change (g/cm^2^) after one year of denosumab therapy at both LS and FN. The group with subsequent injection between five and seven months was considered the reference group. All multivariable regression analyses were adjusted for baseline BMD values, age of the patient, proton pump inhibitors (yes/no), selective serotonin reuptake inhibitors (yes/no), serotonin-norepinephrine reuptake inhibitors (yes/no), tricyclic antidepressants (yes/no), and the number of months between the first and second BMD measurements. All statistical analyses were performed using the SAS/STAT (version 9.2; SAS Institute, Cary, NC, USA) software package running on Windows XP Professional. The criterion for statistical significance was set at *α* = 0.05.

## 3. Results

Out of the 924 charts reviewed, 403 female (92.4%) and 33 male (7.6%) with osteoporosis and on denosumab therapy were eligible. The mean age and standard deviation (SD) of patients were 67.8 (10.7) years. The baseline BMD at the lumbar spine and femoral neck was 0.803 (0.128) and 0.622 (0.099) g/cm^2^. A total of 54.13% of patients had a history of at least one nonvertebral fracture while 23.17% had a history of a vertebral fracture prior to denosumab therapy. Upon completion of the analysis, patients were allocated to one of three groups based on their compliance to denosumab therapy (subsequent injection of denosumab (1) less than five months, (2) between five and seven months, and (3) more than seven months after their initial subcutaneous injection). Demographics are presented in Table 1. Twelve patients received a subsequent injection of denosumab less than 5 months, 365 patients between five and seven months, and 59 patients greater than seven months. Baseline BMD results are presented for each of the defined groups along with the history of nonvertebral and vertebral fractures. Moreover, the frequency of proton pump inhibitors, selective serotonin reuptake inhibitors, serotonin-norepinephrine reuptake inhibitors, and tricyclic antidepressants for each group are reported. Comparison data taken after 1 year of denosumab treatment is presented in Table 2. Descriptive statistics are provided along with the BMD values after one year of denosumab treatment. Incident fracture occurrence is also provided. The mean duration and standard deviation (SD) of treatment from baseline injection were 3.03 months (1.18), 6.05 months (0.38), and 9.43 months (3.06) for patients who received a subsequent injection of denosumab less than 5 months, between 5 and 7 months, and greater than 7 months, respectively.

To compare the change in BMD after one year of denosumab therapy, multivariable regressions were performed for the lumbar spine and femoral neck. Using the group receiving a subsequent injection between 5 and 7 months as a reference, the groups receiving a subsequent injection less than 5 months and greater than 7 months later were analyzed. Results are provided in Table 3. The difference in lumbar spine BMD change (95% confidence interval) after one year was 0.00035 g/cm^2^ (−0.0124, 0.0131) for patients receiving a subsequent injection greater than 7 months and 0.020 g/cm^2^ (−0.0058, 0.0479) for patients receiving a subsequent injection less than 5 months, as compared with the reference group receiving an injection between 5 and 7 months after their initial injection, respectively. The difference in femoral neck BMD change (95% confidence interval) after one year was −0.0054 g/cm^2^ (−0.0171, 0.0063) for patients receiving a subsequent injection greater than 7 months and 0.0079 g/cm^2^ (−0.0165, 0.0324) for patients receiving a subsequent injection less than 5 months, as compared with the reference group receiving an injection between 5 and 7 months after their initial injection, respectively. The relationship between drug administration and change in BMD was not clinically or statistically significant (*p* > 0.05).

## 4. Discussion

The efficacy of a treatment depends on both the effectiveness of the therapy and the adherence to recommended dosage regimens [7]. Osteoporosis, like other chronic medical conditions, must have strategies to maintain patient compliance to therapy. Noncompliance to long-term bisphosphonate or denosumab treatment can result in the decline of BMD values and ultimately result in an increased risk of fracture.

Denosumab is an effective treatment to prevent the progression of osteoporosis in patients. It is a fully human monoclonal antibody that binds to the receptor activator for the RANK ligand, leading to increased bone mineral density and decreased risk of fractures. While the pharmacology of denosumab is ideal, the success of the drug as therapy for osteoporosis hinges on patient adherence to established clinical guidelines. This highlights the importance of identifying reasons for poor adherence and adapting clinical guidelines accordingly.

This study explores the extent to which noncompliance to denosumab can affect the changes in BMD. In this retrospective cohort study, the multivariable regressions revealed that there were no statistically significant differences in the BMD values between patients who received a subsequent subcutaneous injection of denosumab less than 5 months, between 5 and 7 months, and greater than 7 months. However, our study does not suggest that patients and clinicians can extend the duration between subsequent injections of denosumab, since there is a steady decline in drug levels in circulation 6 months following administration, with complete elimination of the drug at the 9-month mark. This study does suggest that, only under unforeseen circumstances, when a patient is otherwise unable to receive a subsequent injection of denosumab in a timely manner, then a delay may be acceptable. Otherwise, all patients should continue to receive their subsequent doses every 6 months, as per the product monograph.

There are factors inherent to the nature and administration of Prolia, as well as factors unique to this patient population, that may explain improved compliance rates. Firstly, it must be acknowledged that denosumab can only be administered through subcutaneous injection by a healthcare professional. Therefore, physicians have significant control over the adherence to denosumab, being able to establish a dosing course, directly monitor adherence to the treatment, and communicate the importance of adherence to patients when doses are missed. Furthermore, there are factors that may explain the higher adherence rates in this study compared to other observational studies. One such reason is that the subjects of the study are recommended to use the ProVital program, a support program aimed at helping patients stay on their dosing schedule. Patients receive a call from a nurse a week before their next scheduled injection as a reminder. Additionally, at our academic center, a specialty pharmacy exists, which also reminds patients about their appointment one week in advance. Together, these two factors have contributed to the high compliance rate for patients in this study and may be significantly higher than many community practices.

There are several limitations to this study. The limited number of patients in the less than 5 months and greater than 7 month groups makes it difficult to draw definitive conclusions. We are also unable to comment on differences in fracture rates between the groups given the small number of events owing to the small size of the cohort and short follow-up duration. A longer follow-up duration in a larger cohort may in fact show a difference in BMD or even fracture rates in patients not compliant to therapy. Furthermore, there is uncontrolled variability among patients in each group as additional medications can influence BMD, and this was not accounted for in the multivariable analysis. Finally, the patients excluded due to a fracture in the femoral neck and lumbar spine during the review period along with a change in DXA machine could affect the results of the investigation.

## 5. Conclusion

Patient compliance is a simple and economic way for patients to benefit from drug efficacy. No matter how effective a drug is, without the proper adherence to the recommended dosing regimens, the patient may not fully benefit from the course of therapy. In summary, our study found that there were no statistically significant differences in BMD values between patients who received a subsequent injection of denosumab less than five months, between five and seven months, and greater than seven months. These findings indicate that clinicians may have some flexibility for administering denosumab if there are extenuating circumstances for which patients must delay their subcutaneous injection. However, the short follow-up duration and small sample size do not allow us to make such a conclusion. Patients should continue to receive their denosumab therapy every six months if this is possible. A follow-up study with a larger sample size and longer follow-up duration will allow for a more in-depth characterization of this relationship.

Furthermore, our study suggests that utilizing different patient adherence programs either at a local or at a national level is an effective method to maintain a high level of patient compliance, thus ultimately improving the real-world effectiveness of this therapy.

## Figures and Tables

**Table 1:** Participant baseline characteristics stratified by time of denosumab injection.

Characteristic	Time of subsequent injection of denosumab		
	<5 months	5–7 months	>7 months
Sample size (*n*)	12	365	59
Age, years, mean (SD)	67.50 (9.59)	68.35 (10.62)	64.34 (11.20)
Women, % (*n*)	91.67% (11)	92.6% (338)	91.53% (54)
Proton pump inhibitor (PPI) use, % (*n*)	27.27% (3)	26.30% (96)	22.03% (13)
Selective serotonin reuptake inhibitor (SSRI) use, % (*n*)	0.00% (0)	6.85% (25)	7.02% (4)
Serotonin-norepinephrine reuptake inhibitor (SNRI) use, % (*n*)	0.00% (0)	1.37% (5)	5.17% (3)
Tricyclic antidepressant (TCA) use, % (*n*)	9.09% (1)	4.11% (15)	5.17% (3)
BMD, mean (SD)			
Lumbar spine	0.850 (0.149)	0.800 (0.131)	0.808 (0.101)
Femoral neck	0.629 (0.043)	0.623 (0.100)	0.619 (0.100)
History of nonvertebral fracture, % (*n*)	41.67% (5)	55.34% (202)	49.15% (29)
History of vertebral fracture, % (*n*)	16.67% (2)	23.56% (86)	22.05% (13)

**Table 2:** Participant characteristics stratified by time of denosumab injection after one-year follow-up.

Characteristic	Time of subsequent injection of denosumab		
	<5 months	5–7 months	>7 months
BMD, mean (SD)			
Lumbar spine	0.909 (0.141)	0.840 (0.137)	0.849 (0.102)
Femoral neck	0.655 (0.080)	0.638 (0.104)	0.635 (0.097)
Incident nonvertebral fracture, % (*n*)	0.0% (0)	2.47% (9)	3.39% (2)
Incident vertebral fracture, % (*n*)	0.0% (0)	0.0% (0)	0.0% (0)

Mean duration of treatment from baseline injection in months (SD)	3.03 (1.18)	6.05 (0.38)	9.43 (3.05)

**Table 3:** Regression analysis comparing change in bone mineral density (BMD) between groups [reference group: 5–7 months]^*∗*^.

	Groups: time between 1st and 2nd injection was	Estimate	95% confidence limits
Lumbar spine	Greater than 7 months	0.00035	−0.0124 to 0.0131
	Less than 5 months	0.020	−0.0058 to 0.0479

Femoral neck	Greater than 7 months	−0.0054	−0.0171 to 0.0063
	Less than 5 months	0.0079	−0.0165 to 0.0324

^*∗*^All multivariable regression analyses were adjusted for baseline BMD values, age of the patient, proton pump inhibitors (yes/no), selective serotonin reuptake inhibitors (yes/no), serotonin-norepinephrine reuptake inhibitors (yes/no), tricyclic antidepressants (yes/no), and the number of months between the first and second BMD measurements.
